# Structural characterization, biofunctionality, and environmental factors impacting rheological properties of exopolysaccharide produced by probiotic *Lactococcus lactis* C15

**DOI:** 10.1038/s41598-023-44728-w

**Published:** 2023-10-19

**Authors:** Gafar Bamigbade, Abdelmoneim H. Ali, Athira Subhash, Camila Tamiello-Rosa, Farah R. Al Qudsi, Gennaro Esposito, Fathalla Hamed, Shao-Quan Liu, Ren-You Gan, Basim Abu-Jdayil, Mutamed Ayyash

**Affiliations:** 1 Department of Food Science, College of Agriculture and Veterinary Medicine, United Arab Emirates University (UAEU), Al Ain, UAE; 2 Department of Food Science, Faculty of Agriculture, Zagazig University, Zagazig, 44511 Egypt; 3 Department of Nutrition and Food Technology, Jordan University of Science and Technology, Irbid, 21121 Jordan; 4 Science Division - New York University Abu Dhabi, NYUAD Campus, Saadiyat Island, PO Box 129188, Abu Dhabi, UAE; 5 Department of Physics, College of Science, United Arab Emirates University (UAEU), PO Box 1555, Al Ain, UAE; 6 Department of Food Science and Technology, Faculty of Science, National University of Singapore, Science Drive 2, Singapore, 117542 Singapore; 7 Singapore Institute of Food and Biotechnology Innovation (SIFBI), Agency for Science, Technology and Research (A*STAR), Singapore, 138669 Singapore; 8 Chemical and Petroleum Engineering Department, College of Engineering, United Arab Emirates University (UAEU), PO Box 15551, Al Ain, UAE

**Keywords:** Biopolymers, Polymer characterization

## Abstract

Exopolysaccharides (EPSs) possess distinctive rheological and physicochemical properties and innovative functionality. This study aimed to investigate the physicochemical, bioactive, and rheological properties of an EPS secreted by *Lactococcus lactis* subsp. *lactis* C15. EPS-C15 was found to have an average molecular weight of 8.8 × 10^5^ Da and was identified as a hetero-EPS composed of arabinose, xylose, mannose, and glucose with a molar ratio of 2.0:2.7:1.0:21.3, respectively. The particle size and zeta potential represented 311.2 nm and − 12.44 mV, respectively. FITR exhibited that EPS-C15 possessed a typical polysaccharide structure. NMR displayed that EPS-C15 structure is → 3)α-d-Glc^vi^ (1 → 3)α-d-Xyl^v^ (1 → 6)α-d-Glc^iv^(1 → 4)α-d-Glc(1 → 3)β-d-Man(1 → 2)α-d-Glc^i^(1 → . EPS-C15 scavenged DPPH and ABTS free radicals with 50.3% and 46.4% capacities, respectively. Results show that the antiproliferative activities of EPS-C15 revealed inhibitions of 49.7% and 88.1% against MCF-7 and Caco-2 cells, respectively. EPS-C15 has antibacterial properties that inhibited *Staphylococcus aureus* (29.45%), *Salmonella typhimurium* (29.83%), *Listeria monocytogenes* (30.33%), and *E. coli* O157:H7 (33.57%). The viscosity of EPS-C15 decreased as the shear rate increased. The rheological properties of the EPS-C15 were affected by changes in pH levels and the addition of salts. EPS-C15 is a promising biomaterial that has potential applications in various industries, such as food, pharmaceuticals, and healthcare.

## Introduction

Exopolysaccharides (EPSs) are large, complex, and high-molecular-weight biological macromolecules composed of repeated monosaccharide units. EPSs are classified into two main categories: Hetero-EPS and homo-EPSs depending on their molar ratios and the type of monosaccharide subunits contained. EPSs consisting of identical mono-sugar subunits are referred to as homo-EPSs, while those with varying building blocks are known as hetero-EPSs^1^. The production of EPSs by various microorganisms, such as microalgae, non-filamentous fungi, and bacteria, particularly lactic acid bacteria (LAB), has been extensively studied^2,3^. It was reported that the characteristics, composition, and structure of EPSs produced by LAB are influenced by the genetic makeup of the bacterial species or strains, as well as the environmental conditions during growth, resulting in different LAB-producing EPSs with distinct pathways and properties^4,5^. For example, *Lactobacillus delbrueckii* subsp. *bulgaricus* and *Streptococcus thermophilus* employ Wzx/Wzy-dependent pathways and extracellular synthetic pathways to produce homo-EPSs and hetero-EPSs, respectively^6^.

EPSs are safe substances, and therefore they are suitable for use in food applications, such as the preservation and enhancement of nutritional and sensory qualities. The growing body of research documenting the various biological activities of microbial EPSs has led to increased interest in their uses across various industries, including foods, pharmaceuticals, cosmetics, and healthcare^7^. These activities have been linked to the physical, chemical, and biological properties of EPSs, which have been shown to exhibit antioxidant, anticancer, and antibacterial properties^8^. Furthermore, the rheological properties of EPSs, such as storage and loss moduli, are considered important parameters during their application in foods and healthcare^9^. Different EPSs have various rheological parameters, and this is determined by the type and substitutions of functional groups, monosaccharide content, molecular weight, and the type of glycosidic bonds^6^. It is a well-established fact that the incidence of non-communicable diseases, such as diabetes and cancer, is increasing globally. The World Health Organization (WHO) predicts that by 2030, the number of global cancer patients will reach 23.6 million, with breast cancer being the most common, although colon cancer will have the highest mortality rate^10,11^. In order to address this public health concern, researchers are actively searching for natural anticancer agents that can mitigate the negative side effects of conventional chemotherapy^12^. Additionally, diabetic patients often experience side effects, such as flatulence and diarrhea, from consuming antidiabetic inhibitors like acarbose, used to control blood sugar levels. Therefore, there is a need for alternative inhibitors of natural origin that do not have known toxic effects^13^.

EPSs produced by LAB offer a variety of antioxidant sources that could be applied in several fields^14^. LAB-EPSs have the possibility of becoming an oral hypoglycemic vehicle to avoid type II diabetes. In addition, EPSs can inhibit α-amylase and α-glucosidase activities, decreasing the rate of food hydrolysis to glucose in the intestine, which, by the way, decreases the content of glucose in the blood^15^. LAB-EPSs are also potential antiproliferative substances that have antiproliferative influences on liver, breast, intestinal, and other cancer cells^16^. Simultaneously, LAB-derived EPSs could be applied as a prebiotic to boost human health via a selective improvement of EPS-producing LAB or other probiotic strains and progress the stress tolerance of EPS-forming LAB^14^. Among the different species of LAB, *Lactococcus lactis* subsp. *lactis* is regularly utilized as an industrial starter culture in the manufacture of various dairy products^17^. Significant progress has been achieved in investigating the synthesis of EPS by various lactic acid bacteria, including *Lactococcus lactis*. Specific *eps* gene clusters have been identified and thoroughly characterized in diverse EPS-producing strains of lactococci. EPS produced by *L. lactis* has been reported to improve the texture properties of the cream cheese^18^. Feng et al.^19^, Feng et al.^20^ have characterized EPS from two different *Lactococcus lactis* strains (EPS-Q9 and EPS-Z2). This indicates the significance of investigating new EPS produced by novel newly isolated *Lactococcus* spp^21^.

Recently, *L. lactis* C15 (accession number KX881768) was isolated from camel milk, and it was identified as LAB with potential probiotic properties*.* Different *L. lactis* strains, such as *L. lactis* KX881782 and *L. lactis* KX881768 exhibited remarkable EPS production^22^. However, the characterization of the secreted EPSs was not investigated in detail elsewhere. Therefore, this study aimed to examine the characteristics of EPS-C15, which is produced by the L. lactis C15 strain, a potential probiotic. The study investigated the physicochemical properties of EPS-C15, as well as its biological activities, including antioxidant activity, cytotoxicity, and antipathogenic activities. Furthermore, the study evaluated the effects of different salts (CaCl2 and NaCl) and pH values (4.0 and 6.0) on the rheological properties of EPS-C15.

## Materials and methods

### Materials

*Lactococcus lactis* C15 was isolated from raw camel milk samples on MRS agar and characterized as potential probiotic bacteria by Abushelaibi et al.^22^. It was stored at − 80 °C in a glycerol solution (50%). M-17 broth (LAB-M Limited, Lancashire, UK) was used to culture *L. lactis* C15, which was sub-cultured several times before the extraction of EPSs. Furthermore, *Staphylococcus aureus* ATCC 15,923, *Salmonella* Typhimurium 02-8423, *Listeria monocytogenes* ATCC 7644, and *Escherichia coli* O157:H7 1934 were obtained from Prof. Richard Holley Laboratory, University of Manitoba, Canada. Trichloroacetic acid (TCA), pullulan standard, xylose, galactose, glucose, arabinose, mannose, 2,2-azino-bis(3-ethylbenzo-thiazoline-6-sulphonic acid (ABTS), 1,1-diphenyl-2-picrylhydrazyl (DPPH), α-amylase enzyme, α-glucosidase enzyme, colon carcinoma (Caco-2) cell line, breast carcinoma (MCF-7) cell line, M17 broth, Brain heart infusion (BHI), Deuterium oxide (D2O), 0.1 M of CaCl_2_, and 0.1 M of NaCl. All chemicals and microbial media were purchased from Sigma-Aldrich, St. Louis, Missouri, USA.

### EPS-C15 isolation and purification

The extraction of EPS from *L. lactis* C15 was carried out in three stages according to the procedure described by Wang et al.^23^. Firstly, *L. lactis* C15 was grown in M-17 broth media containing 20 g/L of sucrose. Then, the bacterial cells were removed through centrifugation and filtration. The M-17 broth was incubated at 25.0 ± 0.1 °C for 48 h and then centrifuged at 4000×*g* for 20 min at 4 °C to obtain a cell-free supernatant. Chilled absolute ethanol was added to the cell-free supernatant to precipitate the EPS-C15 polymer, which was left at 4 °C for 24 h. Then, the crude extract was obtained through centrifugation at 8000×*g* for 30 min at 4 °C. Afterwards, the crude extract was mixed with trichloroacetic acid to reach a final concentration of 8%, for 30 min at room temperature, followed by centrifugation at 9000×*g* for 20 min. Next, the supernatant was dialyzed (MWCO 20 kDa; Slide-A-Lyzer Cassettes, Thermo Fisher Scientific Inc, MA, USA) against dd-water for 48 h at 4 °C. The partially purified EPS was freeze-dried at − 80 °C, and a portion was stored at − 20 °C in a sealed container for further analysis. A sample was scanned for nucleic acid and protein traces by using a UV–Vis spectrometer (Epoch2, Bio-Tek, VT, USA) at 260 and 280 nm, respectively. No absorbance was detected, indicating the absence of nucleic acids or proteins in the purified EPS-C15.

### EPS-C15 characterization

#### Average molecular weight and monosaccharides composition

The average molecular weight of the extracted EPS-C15 was determined by using gel permeation chromatography attached to a refractive index detector (Waters Cooperation, Herts, UK). The molecular weight was estimated by plotting pullulan as an external standard with molecular weights of 5 kDa, 10 kDa, 20 kDa, 50 kDa, and 100 kDa. The detailed procedure was described by Kansandee et al.^24^. The identification of EPS-C15 monosaccharide residues was performed using the traditional acid hydrolysis method. The acid hydrolysis was performed by mixing EPS-C15 with 5 mL of 2.0 M trifluoroacetic acid, followed by heating at 120 °C for 2 h. The analysis of the monosaccharide composition of EPS-C15 was performed by using the gas chromatography-flam ionization detector (YL6500) supplemented with an HP-Ultra-2 column (250 mm × 0.20 mm × 0.11 μm) according to the method described by Gao et al.^1^. A standard mixture composed of xylose, galactose, glucose, arabinose, and mannose monosaccharides was applied for the identification of monosaccharide profile.

#### Fourier transform-infrared spectroscopy (FTIR)

The analysis of the functional groups in the purified EPS-C15 was carried out by using an attenuated total reflectance (ATR-FTIR) spectrophotometer (Perkin-Elmer Inc., Norwalk, CT, USA) in the spectrum range of 4000–400 cm^−1^ at room temperature. In addition, a diamond/ZnSe crystal plate was employed, and EPS-C15 powder was positioned on the plate.

#### Nuclear magnetic resonance (NMR)

The NMR analysis of EPS-C15 was performed by using an Avance III Bruker spectrometer (Bruker Corporation, MA, USA) equipped with a cryo-probe and z-axis gradient and operating at 600.19 and 150.92 MHz for ^1^H and ^13^C. All spectra were acquired at 298 K with a single sample prepared with 10 mg of product dissolved in 550 µL D_2_O (Sigma). A general polysaccharide characterization procedure as reported by Bubb^25^ was followed with one-dimensional (1D) and two-dimensional (2D) spectra (^1^H and ^13^C) used to determine the homonuclear and heteronuclear correlation pattern. Further experimental details are given in Supplementary Materials. The CASPER program (www.casper.organ.su.se/casper) was applied to infer structural conclusions from the NMR as described by El-Deeb et al.^26^.

#### Scanning electron microscopy (SEM)

The surface morphology of EPS-C15 was assessed using scanning electron microscopy (SEM, Akishima, Tokyo, Japan) by operating at an accelerating voltage of 20 kV. The sample (5 mg) was air-dried and sputter-coated with gold using a Cressington 108 Auto Sputter Coater (Ted Pella Inc., Redding, CA, USA).

#### Thermal properties

A differential scanning calorimeter (DSC 25, TA instrument, DE, USA) was used to determine the thermal behavior of EPS-C15 under changes in temperature. An amount of 25 mg of EPS-C15 sample was heated in an alumina crucible from 20 to 350 °C, and an empty pan was applied as a reference. The thermogram was recorded following the protocol of Sasikumar et al.^27^. The DSC was repeated in triplicates.

#### Zeta potential and particle size analysis

Zeta potential is used to characterize the surface charge distribution and stability of EPSs. The Nano Plus Zeta potential and particle size analyzer (Particulate System, GA, USA) were utilized for the analysis of the zeta potential and particle size of EPS-C15 (0.5% w/v). The analysis was done as per the procedure reported by Sasikumar et al.^27^.

### Evaluation of EPS-C15 bioactivities

#### Antioxidant capacity

The ability of EPS-C15 to exert antioxidant activity was evaluated by in vitro ABTS and DPPH radical scavenging assays. For DPPH, A 800 μL of the DPPH reagent (0.1 mM DPPH dissolved in 95% methanol) was added to 200 μL of EPS solution in glass test tubes. The mixture was shaken vigorously and incubated in the dark at room temperature for 30 min. Methanol was used as a blank. The absorbance of the incubated samples was measured at 517 nm^28^. For ABTS, A stock solution of ABTS was prepared by mixing stock solutions of 7.4 mM ABTS aqueous solution and 2.6 mM potassium persulphate aqueous solution in equal quantities (molar ratio = 1:0.35) and allowing them to react for 12 h in the dark at room temperature. A fresh ABTS reagent was prepared by mixing 1 mL of ABTS^•+^ solution with 50–60 mL of the buffered methanol to obtain an absorbance of 0.70 ± 0.02 at 734 nm after equilibration at 30 °C. Twenty microliters of EPS solution was added to 2 mL of an ABTS reagent and incubated at 30 °C for 6 min. The absorbance of the mixture was measured at 734 nm^28^. The percentage of scavenging activity was assessed at two different concentrations of EPS-C15 (5 and 10 mg/mL) dissolved in dd-water^9^. The percentage of DPPH and ABTS scavenging activities was calculated using the following equation:

1 $$\mathrm{Scavenging \;Rate }\left(\mathrm{\%}\right)=\left[1-\frac{{\mathrm{A}}_{\mathrm{sample}-}{\mathrm{A}}_{0}}{{\mathrm{A}}_{\mathrm{control }- }{\mathrm{A}}_{0}} \right]\times 100$$

where A_0_ is the blank absorbance (negative control), and A_control_ refers to the absorbance of the radical (positive control).

#### Antidiabetic activity

Following the detailed procedure described by Ayyash et al.^12^, the inhibition of α-amylase and α-glucosidase was assessed by using two different concentrations (100 and 200 µg) of EPS-C15. The following equation was applied to calculate the inhibition percentages of α-amylase and α-glucosidase.

2 $$\mathrm{Inhibition }\left(\mathrm{\%}\right)=\left(1-\frac{{\mathrm{Abs}}_{\mathrm{sample}-}{\mathrm{Abs}}_{\mathrm{blank}}}{{\mathrm{Abs}}_{\mathrm{control }}} \right)\times 100$$

#### Cytotoxic activity

Colon carcinoma (Caco-2) and breast carcinoma (MCF-7) cell lines were used to assess the cytotoxic activity of EPS-C15 at two concentrations (5 and 10 mg/mL). The protocol used was described in detail by Ayyash et al.^9^, and the percentage of cytotoxicity was calculated according to the following equation:

3 $$\mathrm{Cytotoxicity }\left(\mathrm{\%}\right)=\left[1-\frac{{\mathrm{R}}_{\mathrm{sample}-}{\mathrm{R}}_{0}}{{\mathrm{R}}_{\mathrm{control }- }{\mathrm{R}}_{0}} \right]\times 100$$

where R_sample_ is the absorbance ratio of OD570/OD605 of the EPS, R_control_ is the absorbance of the control sample (without EPS), and R_0_ is the average background considered a negative control with no cells.

#### Antibacterial activity

EPS-C15 was assayed for antibacterial activity against four foodborne pathogenic bacterial strains, including *S. aureus* ATCC 25923, *S.* Typhimurium 02-8423, *L. monocytogenes* ATCC 7644*,* and *E. coli* O157:H7. The experiment was performed by assessing the inhibition of bacteria as the EPS-C15 concentration increased following the detailed method described by Jeong et al.^29^. Briefly, 100 μL of EPS-C15 aqueous solution (5 mg/mL) was added to the culture activated in brain heart infusion (250 μL), and then incubated for 18 h at 37 °C. Afterwards, the pathogens were enumerated on brain heart infusion agar and incubated for 24 h at 37 °C.

### Rheological properties

After the extraction of EPS-C15, the rheological tests were performed using a Discovery Hybrid Rheometer (TA Instruments, New Castle, DE, USA). Three aqueous solutions (5 mg/mL) of EPS-C15 were prepared by using 0.1 M of CaCl_2_, 0.1 M of NaCl, and distilled-deionized water. In addition, the three solutions were prepared at two pH values (4.0 and 6.0). The rheological properties of the aqueous solutions were determined using cone and plate geometries (1 cone angle, 50 mm gap, and 50 mm diameter). The temperature of the plate was maintained at 25.0 ± 0.1 °C.

#### Apparent viscosity

The flow curves of the extracted EPS-C15 solutions were measured upward and downward at a shear rate ranging from 10^1^ to 1000 s^–1^, with all the tests performed at 25.0 ± 0.1 °C. The area between the upward and downward curves was used to evaluate the degree of thixotropy. In addition, the flow curves of EPS-C15 were described using the power law model (Eq. 4).

4 $$ \tau = m\dot{Y}{\upeta} $$

where $\tau$ is the shear stress, $m$ is the consistency coefficient, $\dot{Y}$ is the shear rate, and ƞ is the flow behavior index.

#### Temperature-dependent behavior

The dependence of the apparent viscosity of EPS-C15 on temperature was assessed by raising the temperature up to 80 °C at a heating rate of 3 °C/min at a fixed shear rate of 20 s^–1^. The flow activation energy of EPS-C15 was calculated by using Eq. 5 (Arrhenius equation).

5 $$\mathrm{n}={n}_{0}e\frac{{E}_{a}}{RT}$$

where $\mathrm{n}$ refers to the apparent viscosity, ${n}_{0}$ is the Arrhenius constant, ${E}_{a}$ is the flow activation energy, *R* is the gas constant, and *T* is the temperature in Kelvin.

#### Amplitude and frequency sweep tests

The amplitude test was performed to determine the linear viscoelastic region of EPS-C15. An amplitude strain sweep was carried out at a constant frequency of 1 Hz over a strain range of 0.1–20%. On the other hand, the frequency sweep test was conducted in the frequency range of 0.1–20 Hz at a constant strain within the linear viscoelastic region (< 2%) to determine the viscoelastic behavior of EPS-C15, including the storage modulus (G′), loss modulus (G″), and the loss tangent (tan δ). All amplitude and frequency sweep tests were carried out at a constant temperature of 25.0 ± 0.1 °C.

#### Thixotropic behavior

Oscillation step tests were used to evaluate the breakdown of the inner structure of EPS-C15 under high shearing conditions and its subsequent recovery. G′ and G″ moduli were measured in an oscillation-time test at a frequency of 1.0 Hz with the following three test intervals: the first-time segment was 400 s at a stress of 0.2 Pa (to simulate behavior at rest within the linear viscoelastic range), the second segment was 60 s at a stress of 50 Pa (to simulate structural breakdown of EPS-C15 during application at high strain far beyond the linear viscoelastic range), and the third was 200 s at a stress of 0.2 Pa (to simulate structural regeneration at rest).

### Statistical analysis

The different measurements were carried out in triplicate, and one-way analysis of variance (ANOVA) test was used to compare the differences. In addition, the quantitative data were expressed as mean ± standard deviation with *P* < 0.05 considered statistically significant. Moreover, Fisher’s test was applied to compare mean values at the level of significance.

## Results and discussion

### Molecular weight and monosaccharide composition of EPS-C15

EPSs chemical composition varies between species and strains, which, by the way, influences their properties and potential applications^6^. The results obtained by gel permeation chromatography and refractive index analysis showed that the partially purified EPS-C15 had an average molecular weight of 8.8 × 10^5^ Da (Fig. S1), which was higher than the molecular weight of the EPS partially purified from *L*. *bulgaricus* (5.37 × 10^4^ Da)^11^. It was reported that the molecular weight of LAB-EPSs was in the range between 123.84 and 178.72 kDa^30^. According to the chemical composition, EPSs produced by *L. lactis* strains can be classified as neutral or acidic. Neutral EPSs are composed of two or more different monosaccharide units, but neither organic acids nor acidic EPSs present uronic acid groups. The monosaccharide composition analysis revealed that EPS-C15 is composed of arabinose, xylose, mannose, and glucose at a molar ratio of 2.0:2.7:1.0:21.3, respectively (Table S1). EPS produced by *L. lactis* IMAU11823 comprised glucose and mannose with a molar ratio of 7.01:1.00, and a molecular weight of 6.10 × 10^5^ Da, while another EPS produced by the same strain was composed of mannose, glucose, and rhamnose at molar ratios of 7.45:1.00:2.34, and a molecular weight of 2.93 × 10^5^ Da ^31^. Furthermore, it was reported that ribose, fucose, mannose, glucose, galactose, and arabinose with a molar ratio of 5.48:0.39:9.77:4.03:1.00:1.92, respectively were the main monosaccharides of an EPS extracted from *L. lactis*^32^. Another study reported that mannose, ribose, rhamnose, glucuronic acid, glucose, galactose, xylose, and arabinose, representing 3.97, 4.83, 3.59, 6.83, 44.10, 23.91, 6.34, and 6.43, respectively, were the predominant monosaccharides in the EPS produced from *L. lactis*^30^*.* Glucose, which positively affects the viscosity of EPSs, is commonly found in their monosaccharide composition. Moreover, the presence of galactose, glucuronic acid, mannose, arabinose, rhamnose, fucose, xylose, and fructose in EPSs has been reported^33^.

### FTIR and NMR

**Figure 1:**
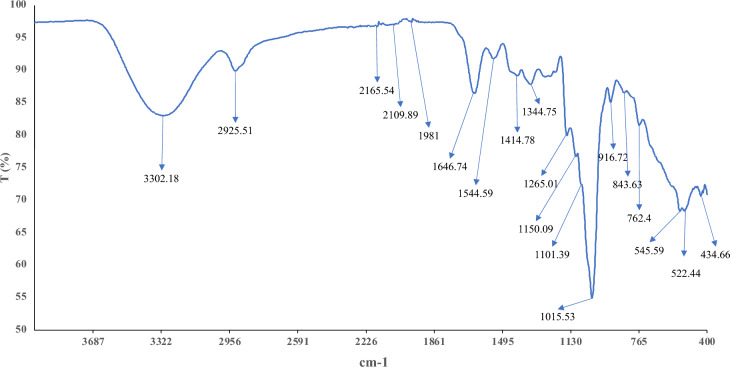
FTIR spectrum of EPS-C15 at wavenumbers range of 4000–400 cm^–1^.

The infrared spectrum of the functional group analysis obtained by the ATR-FTIR is elucidated in Fig. 1. It was shown that the absorption bands at 843.63 cm^−1^ and 916.72 cm^−1^ could be assigned to the α- and β-configurations of the sugar units^34^. In addition, the band at 3302.18 cm^−1^ was the ideal stretching vibration of the hydroxyl group. The elongating vibration of the C–H (asymmetrical) of the aliphatic CH_2_ group was assigned to 2925.51 cm^−1^^35^. The 1646.74 cm^−1^ band was attributed to the stretching vibration of a carboxyl group^36^. Furthermore, the region of 900–1200 cm^−1^ was attributed to the vibrations of the C–O–C glycosidic linkages. This region is described as the fingerprint of EPSs^34^. The band at 1414.78 cm^−1^ was ascribed to the bending vibrations of the O–H bond. It is worth noting that the absence of 1730 and 1720 cm^−1^ bands implies that EPS-C15 does not encompass uronic acid^37,38^.

**Figure 2:**
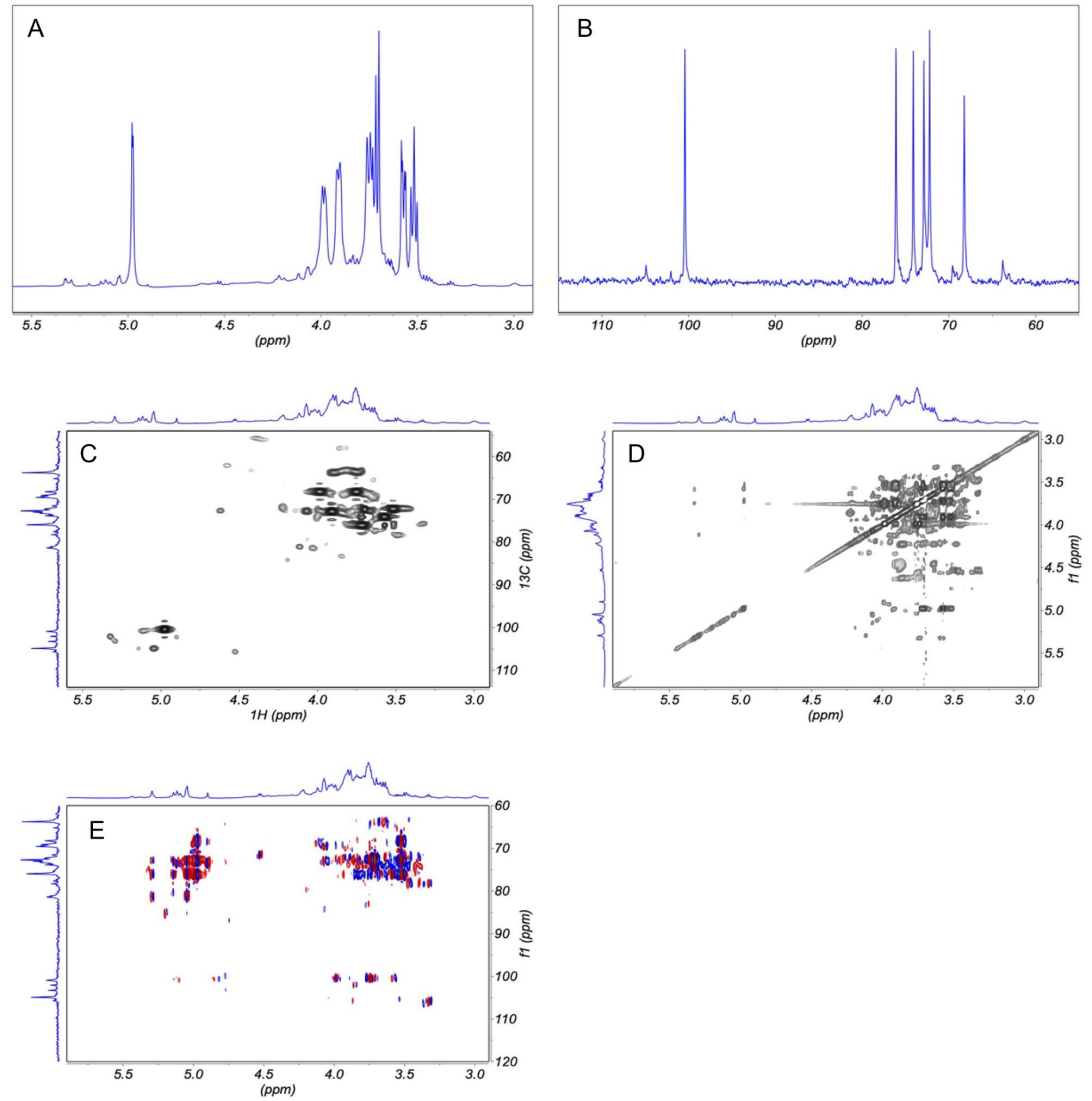
1D and 2D NRM analysis of EPS-C15. (**A**) Region from 1D ^1^H spectrum. (**B**) Region from 1D ^13^C spectrum. (**C**) Region from 2D ^1^H TOCSY. (**D**) Region from 2D ^13^C-^1^H HSQC spectrum. (**E**) Overlay of 2D ^13^C-^1^H HSQC (black contours) and 2D ^13^C-^1^H HMBC (red-blue contours).

Figure 2 groups the NMR evidence obtained for EPS-C15, namely the 1D ^1^H (panel A), ^13^C (panel B) traces, and the 2D ^13^C-^1^H HSQC (panel C), 2D ^1^H‒^1^H TOCSY (panel D) maps. An overlay of 2D ^13^C-^1^H HMBC and 2D ^13^C-^1^H HSQC is also shown (Fig. 2E). The peaks occurring in the region from 3 to 4 ppm in the ^1^H NMR spectrum arise from the sugar protons H-2, H-3, H-4, H-5, and H-6. The anomeric proton and carbon resonances typically occur in the regions 4.7–5.5 ppm of ^1^H spectra and 95.0–110.0 ppm of ^13^C spectra, respectively ^35^. Assignments were carried out based on the connectivity pattern that could be established from the analysis of the TOCSY and HSQC maps. The consistency of the interpretation was further controlled by comparing the long-range relayed correlation of the HMBC map to the corresponding HSQC and TOCSY connectivities (Fig. 2E). The experimental information was then matched with the CASPER software^39^ computation to obtain identification and configuration of the polysaccharide units. The pattern of α-configurations of D-glucopyranose and D-xylopyranose and β-configurations of D-mannopyranose could be assessed. The CASPER attribution of the experimental anomeric CH correlations obtained from ^1^H-^13^C NMR HQSC is presented in Table 1. Based on the computation results, the proposed backbone structure of EPS-C15 is: → 3)α-d-Glc^vi^ (1 → 3)α-d-Xyl^v^ (1 → 6)α-d-Glc^iv^(1 → 4)α-d-Glc^iii^(1 → 3)β-d-Man^ii^(1 → 2)α-d-Glc^i^(1 → . It is assumed that arabinose is attached to the terminals^37^. The ^13^C NMR spectrum did not exhibit any peak at δ > 170 ppm, which denotes the lack of uronic acid function^37^.

**Table 1:** ^1^H and ^13^C chemical shift assignments of EPS-C15.

Residues	NMR	Chemical shifts (ppm)^a^					
		1	2	3	4	5	6,6′
→ 2)α-d-Glc^i^(1 →	^1^H	111.86	82.53	76.99	70.45	85.32	61.47
	^13^C	5.57	3.95	4.11	3.50	4.48	3.83,4.00
→ 3)β-d-Man^ii^(1 →	^1^H	103.13	95.28	83.20	n.d	78.26	63.07
	^13^C	4.98	4.64	3.84	3.97	3.57	3.98,4.08
→ 4)α-d-Glc^iii^(1 →	^1^H	104.85	75.96	87.44	79.91	102.89	61.56
	^13^C	5.18	3.93	4.66	3.72	4.45	3.99,4.06
→ 6)α-d-Glc^iv^(1 →	^1^H	102.26	76.68	77.74	69.57	88.89	68.23
	^13^C	5.32	3.95	4.03	3.53	4.43	3.90,3.98
→ 3)α-d-Xyl^v^(1 →	^1^H	100.40	72.22	105.74	69.09	63.79	ND
	^13^C	5.14	3.65	4.18	4.04	3.59	3.61
→ 3)α-d-Glc^vi^(1 →	^1^H	104.91	84.23	85.38	70.56	84.42	61.28
	^13^C	5.29	4.19	4.06	3.72	4.57	3.92,4.02

^a^Chemical shift assignment by CASPER.

### SEM, thermal properties, zeta potential, and particle size

#### SEM and thermal properties

**Figure 3:**
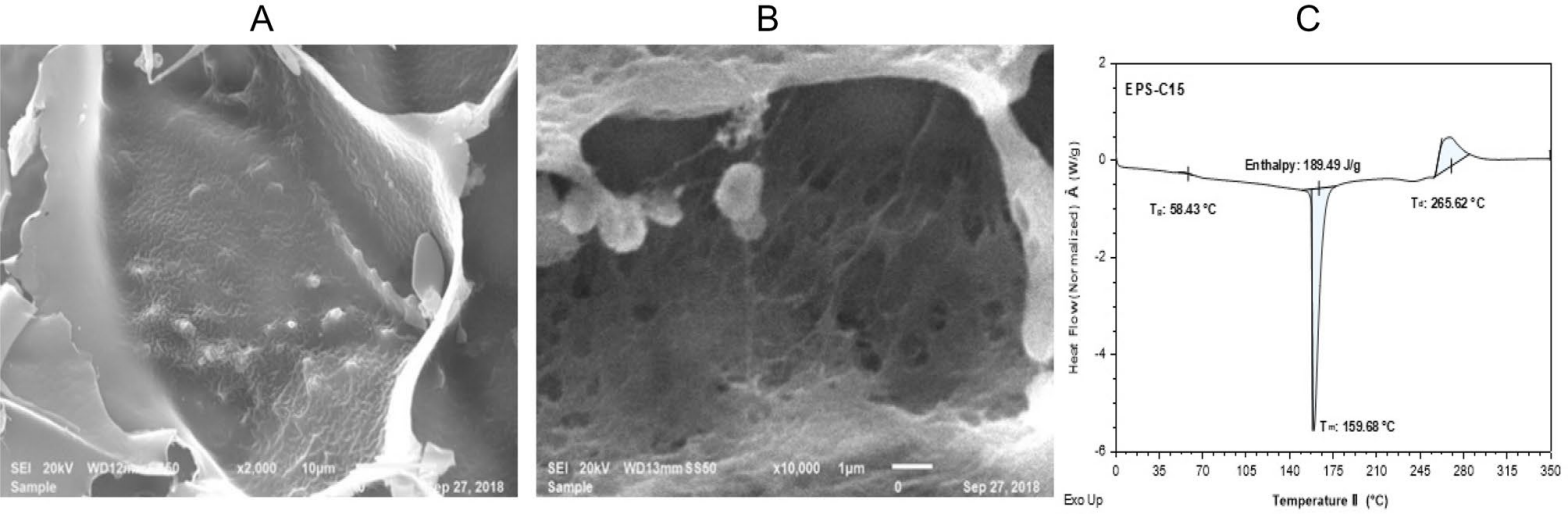
SEM images at magnifications 1000× (**A**) and 1800× (**B**) and DSC thermogram (**C**) of EPS-C15.

The microstructure morphology of EPS-C15 at different magnifications is shown in Fig. 3A,B. The SEM image exhibited a porous structure of EPS-C15 and a flake-like basic configuration. A similar structure was observed for the EPS extracted from the bacterial strain *L. lactis* F-mou isolated from Sahrawi camel milk^33^. The porous network is commonly attributed to *L. lactis* colonies and may contribute to the retained extra moisture, which is a beneficial characteristic for food production, such as cheese-making^34^. The thermal behavior of EPS-C15 analyzed by the DSC (Fig. 3C) exhibited two endothermic peaks and one exothermic peak. The thermal analysis demonstrated that EPS-C15 exhibited two endothermic peaks at 58.43 °C accredited to the glass transition (T_g_) and 159.68 °C corresponding to the melting point (T_m_). The degradation of EPS-C15 occurred at 265.62 °C, represented by the exothermic peak. The higher values of T_g_ and T_m_ suggested that EPS-C15 was stable at elevated temperatures, indicating an advantage to thermal processes in the food processing industries.

#### Zeta potential and particle size

The values of zeta potential and particle size of EPS-C15 were − 12.44 mV and 311.2 nm, respectively. The zeta potential value predicts the colloidal suspension stability of a solution. For better physical colloidal stability, an adequate zeta potential value ranges between − 30 and 30 mV^34^. It was reported that the average zeta potential of two EPSs secreted by the *L. lactis* IMAU11823 strain represented − 3.26 mV and − 9.10 mV, respectively. The two EPSs exhibited diverse particle size distributions. The first EPS showed a single and uneven particle size distribution, mostly in the range between 100 and 1000 nm. The second EPS was mainly distributed in the range of 15–50 nm and 3000–7000 nm, characterized by two comparatively narrow but even peaks. Overall, higher zeta potential values are associated with better solution stability. Accordingly, the variations in zeta potential and particle size distribution among EPSs from different LAB suggested clear variations in their chemical and physical nature^31^.

### Bioactive properties of EPS-C15

#### Antioxidant capacity

**Figure 4:**
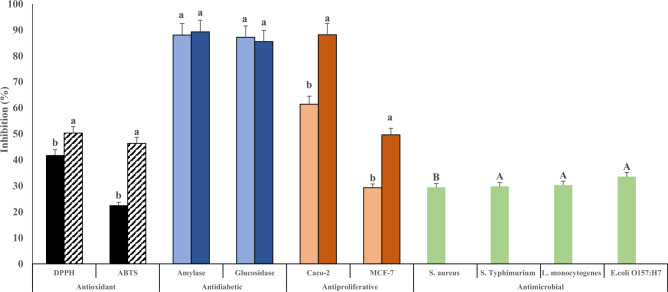
Bioactivities of EPS-C15. Antioxidant [5 mg (black square) and 10 mg (striped square)], antidiabetic [100 µg (light blue square) and 200 µg (dark blue square)], antiproliferative activities [5 mg (orange square) and 10 mg (brown square)], and antibacterial activities [5 mg/mL (green square)]. Bars are means ± standard deviation (n = 6).

The EPS-C15 produced by *L. lactis* C15 showed a potent antioxidant capacity. The results revealed that at 5 mg/mL and 10 mg/mL of EPS-C15, DPPH results were 41.8% and 22.5%, respectively, and the ABTS results represented 50.3% and 46.4%, respectively (Fig. 4). The antioxidant activities were elevated by increasing the level of crude EPS from *L. lactis* IMAU11823, implying a dose-dependent correlation^31^. It was reported that polysaccharides derived from *L. lactis* exhibited antioxidant capacities, which can scavenge DPPH radicals, hydroxyl radicals, H_2_O_2_, peroxyl radicals, superoxide radicals, alkyl radicals, ABTS radicals, and exhibit a reducing power. The composition and the molar ratio of monosaccharides, as well as the types of the glycosyl linkages are crucial in articulating the antioxidant properties of EPSs^1^. Glucose 1 → 6 and arabinose 1 → 4 were closely related to DPPH radical scavenging effect^40^. The antioxidant capacity of EPSs appears to correlate with the monosaccharide’s composition. This is in consonance with the results of Al-Nabulsi et al.^11^ documenting the antioxidant capacity of EPS-L secreted by *L. bulgaricus* from labneh. The antioxidant activities of biomolecules depend on their chemical and physical structure; hence, it would be required to explain the antioxidant activity of biomolecules according to their structural properties. Moreover, monosaccharides profile and functional groups are the main key factors for the antioxidant activity of EPSs^14^.

EPS partially purified from M-17 broth culture of *L. lactis* isolated from milk sources showed strong DPPH capacities at different doses (5–7.5 mg/mL), and it was observed that the extracted EPS exhibited a dose-dependent scavenging capacity. In addition, *L. lactis* F-mou EPS isolated from Sahrawi camel milk exhibited DPPH scavenging capacities at all assayed concentrations (1–4 mg/mL), and as observed, the antioxidant efficiency was EPS dose-dependent^33^. It was reported that the EPS secreted by *L. lactis* NCR112 isolated from *Phyllanthus urinaria* showed a DPPH scavenging capacity of 52.86% at 10 mg/mL^41^. It was also observed that the EPS produced by *L. lactis* 12 isolated from Chinese pickled cabbage displayed in vitro scavenge abilities against hydroxyl and superoxide anion radicals and significantly reduced the malondialdehyde level, while raising the activity of catalase and superoxide dismutase in mice in a dose-dependent manner^42^.

#### Antidiabetic activity

The results show that EPS-C15 demonstrated strong α-amylase and α-glucosidase inhibitory activities (Fig. 4). The inhibition of α-amylase represented 88 and 89.3%, while α-glucosidase inhibition was 87.2 and 85% at a concentration of 100 and 200 µg of EPS-C15, respectively. Glucosidase inhibitors play an important role in diabetes management, and they have been used in the suppression of post-prandial glucose levels in diabetic patients. These inhibitors are widely distributed in living organisms and act by inhibiting enzymes involved in glucose metabolism, leading to a reduction in starch hydrolysis. *L. lactis* antidiabetic effects have been tested, but so far, the inhibitory effects of the EPS secreted by *L. lactis* against α-amylase and α-glucosidase activities have not been described^28^.

#### Antiproliferative activity

The effects of probiotics on different types of cancer cell lines have been reported^43^. Polysaccharides can act as anticancer agents through a broad spectrum of cellular mechanisms, and it was frequently observed that the antiproliferative effects were attributed to the presence of probiotics. As shown in Fig. 4, the concentrations of 5 and 10 mg of EPS-C15 were tested against Caco-2 (human epithelial colorectal adenocarcinoma) and MCF-7 (human breast cancer) cell lines. The results show that the antiproliferative effect against Caco-2 and MCF-7 cell lines, respectively, represented 61.4% and 29.3% of inhibition with 5 mg of EPS, and 88.1% and 49.7% inhibition with 10 mg of EPS. It was reported that the EPS derived from *L. lactis* subsp. *lactis* demonstrated antiproliferative effects by triggering cellular mechanisms leading to cell death in MCF-7 cells^32^. Similarly, 10 mg/mL of the EPS secreted by *L. lactis* NCR112 isolated from *P. urinaria* expressed 86.86% and 50.36% cytotoxic effects against HeLa and HepG2 cell lines, respectively^41^.

#### Antimicrobial activity

EPSs have been stated to comprise different functional groups, such as phosphate, hydroxyl, and carbonyl groups, which were proposed to act a vital role in delivering antimicrobial features^44^. EPS-C15 displayed a significant antimicrobial behavior against all the tested foodborne pathogens (Fig. 4). Among the tested pathogenic bacteria, the extracted EPS showed the highest bactericidal effect against *E. coli* O157:H7 (33.6%), followed by *L. monocytogenes* (30.3%), *S*. Typhimurium (29.8%), and *S. aureus* (29.5%). EPS-C15 had the least significant effect (p < 0.05) on *S. aureus*, which could be attributed to the bacteria's resistance to EPS. Antimicrobial effects were also observed in the EPS produced by *L. lactis* F-mou strain, which possessed a great inhibition against the gram-positive bacteria^33^. EPSs could promote secondary metabolites aggregation in the growth media, which might negatively influence pathogens' growth^45^. Furthermore, EPS can sequester essential nutrients required for microbial growth, thereby limiting the proliferation of potential pathogens. Additionally, some EPS possess direct antimicrobial properties, often attributed to their unique structural characteristics and charge distributions, which can disrupt microbial cell membranes or interfere with essential cellular processes. In addition, EPS could break down the genetic materials (DNA and RNA) inside pathogenic cells^46^. Similarly, the functional groups present in EPSs structure might interact with the microbial cell membranes, yielding antimicrobial actions^14^.

### Rheological properties

#### Apparent viscosity and flow curves of EPS-C15

**Figure 5:**
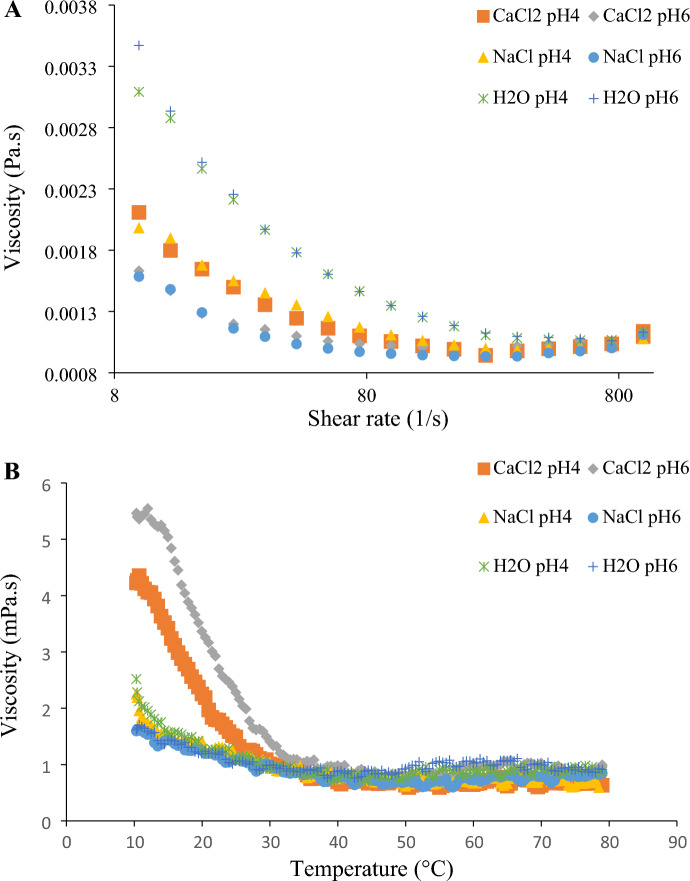
Apparent viscosity (**A**) and temperature-dependent viscosity behavior (**B**) of EPS-C15 in CaCl_2_ at pH 6.0 (diagonal), CaCl_2_ at pH 4.0 (square), NaCl at pH 6.0 (circle), NaCl at pH 4.0 (triangle), H_2_O at pH 6.0 (+), H_2_O at pH 4.0 (times symbol). The tests were repeated in duplicates.

The dependence of the apparent viscosity of EPS-C15 on the shear rate is presented in Fig. 5A. It is evident that EPS-C15 solutions showed higher viscosity, especially at low shear rates. The addition of NaCl or CaCl_2_ to EPS-C15 solutions caused a reduction in the apparent viscosity. The lowest viscosity of EPS-C15 was recorded in acidic salt solutions (pH 4.0). This finding implies that the two cations (Ca^2+^ and Na^+^) might have adverse effects on the viscosity of the EPS-C15-containing product. The decrease in EPS-C15 viscosity can be attributed to the screening effect of the electrolyte on the inter-molecular H-bonding, which might cause a decrease in the three-dimensional network strength^47^. Moreover, the intra-molecular electrostatic repulsions among negatively charged groups in EPS-C15 molecules can contribute to this viscosity reduction by curling polymer coils^47^.

On the other hand, the effect of pH on the viscosity was more pronounced than the effect of salt types, as EPS solutions at pH 6.0 showed higher viscosity than the solutions adjusted at pH 4.0, which can be attributed to the increase of the ionization degree of carboxyl groups in polysaccharide molecules with increasing pH value. This finding agrees with those of Li et al.^48^, who reported higher viscosity of EPS-POS16 at pH 6.0 to 9.0. The decrease in the viscosity of EPS-C15 solutions with increasing electrolyte concentration has been reported for a number of EPSs^48^. It should also be mentioned that the viscosity of EPS-C15 displayed a similar apparent viscosity behavior to other EPSs studied under the same conditions, such as EPS-C47^7^.

Figure S2 shows that all EPS-C15 solutions exhibited a shear thinning behavior as the apparent viscosity decreased with the shear rate. The flow behavior index values obtained from fitting the flow curves with the Power law model (Fig. S2, Table S2) demonstrated that EPS-C15 in water had the highest deviation from Newtonian behavior (ƞ = 1). In contrast, the lowest deviation was detected in samples in electrolyte solutions at pH 6.0. It should be noted that the area of hysteresis loops formed from the upward and downward flow curves was very small for all samples within the experimental errors. This indicates that EPS-C15 exhibited a time-independent behavior under the conditions of the rheological property measurements.

Figure 5B shows the dependence of the apparent viscosity of EPS-C15 on temperature. At a low temperature (25 ± 0.1 °C), the apparent viscosity of EPS-C15 in CaCl_2_ solution at pH = 6.0 was the highest, followed by the same solution at pH 4.0. However, the apparent viscosity of both samples decreased at a temperature higher than 40 °C. This resulted from the effect of the thermal energy on the weak intermolecular forces between EPS-C15 molecules. The differences in the apparent viscosity of different EPS-C15 solutions could not be discerned at temperatures greater than 40 °C. Furthermore, the activation energy of flow presented in Table S3 and determined by Arrhenius’ equation ranged from 10.1 to 33.7 kJ/mol. The highest and lowest activation energy was observed in EPS-C15 in CaCl_2_ solution (pH 4.0) and water (pH 6.0), respectively. Overall, the pH did not show significant influences on the activation energy.

#### Viscoelastic behavior of EPS-C15

The amplitude sweep test was conducted to determine the limit of the linear viscoelastic region for EPS-C15. As shown in Fig. S3 (a and b), the linearity limit for G′ and G″ moduli of all EPS-C15 solutions was less than 2.0% strain. Therefore, the frequency sweep tests of EPS-C15 were carried out at 0.8% strain to avoid mutilation of the structure of the sample during the test. As evident in Fig. S4, the viscous behavior of EPS-C15 solutions was dominant over the elastic behavior at a frequency less than 3.0 Hz. EPS-C15 in CaCl_2_ solution at pH 4.0 was an exception, where the elastic modulus was more significant than the viscous modulus over the entire frequency range tested. The same sample showed the highest values for both elastic and viscous moduli. Moreover, G′ and G′′ of this sample were relatively independent of frequency, opposing the behavior of most EPS-C15 solutions, where both moduli increased with frequency. This behavior can be attributed to the cross-linking effects of cations (Ca^2+^) with the carboxyl groups in EPS-C15.

#### Thixotropic behavior of EPS-C15

In addition to the flow curves hysteresis test, the oscillation step test was performed to evaluate the thixotropic behavior of EPS-C15 solutions. Figure S5 reveals that all EPS-C15 solutions exhibited a time-dependent behavior, as G′ and G′′ moduli values returned back approximately to their original values after releasing the high stress applied. This indicates that the rate of rebuilding the structure in EPS-C15 samples was equivalent to the rate of structure breaking upon the shearing process. Overall, EPS-C15 recovery rates were not affected by the addition of salts. The results of the oscillation step test were in consonance with the flow curves hysteresis test. These findings agree with the rheological properties reported for the EPSs secreted by *Porphyridium sordidum* and *Porphyridium purpureum* in mono and divalent salt solutions^49^.

## Conclusions

An exopolysaccharide (EPS-C15) was biosynthesized by *L. lactis* C15. The partially purified EPS-C15 was characterized by FTIR, NMR, and SEM techniques and examined for its physicochemical, biological, and rheological properties. The structural analysis revealed that EPS-C15 was mainly composed of α-1,3 and α-1,6 linked D-glucopyranose units with an average molecular weight of 8.8 × 10^5^ Da. The porous network structure, which is specific to EPS from *L. lactis*, and its excellent rheological properties strongly support the potential use of EPS-C15 in the food and pharmaceutical industries. EPS-C15 exhibited radical scavenging activities against DPPH (41.8 and 50.3%) and ABTS (22.5 and 46.4%) at 5 and 10 mg/mL, respectively. The antiproliferative activity increased by increasing the concentration of EPS-C15 from 5 to 10 mg, representing 61.4 and 88.1% against Caco-2 and 29.3 and 49.7% against MCF-7, respectively. Furthermore, the type of salt and variations in pH values significantly influenced the rheological characteristics of EPS-C15. EPS-C15 exhibited significant biological activities, including antibacterial, antioxidant, antitumor, and antidiabetic activities, and was highly thermally stable. Therefore, EPS-C15 could be considered a potential biomaterial in the healthcare industry and in thermal processing in the food industry. Further investigation, which may include extensive clinical trials, is required to verify the purported health benefits of EPS-C15. It would be highly advantageous to explore the effects of EPS in realistic scenarios, such as food matrices.

### Supplementary Information

Supplementary Information.

## Data Availability

All data generated or analysed during this study are included in this published article and its supplementary information files.
