# Supplementary material for: Structural characterization, biofunctionality, and environmental factors impacting rheological properties of exopolysaccharide produced by probiotic Lactococcus lactis C15
Source: Sci Rep. 2023 Oct 19;13:17888. doi: 10.1038/s41598-023-44728-w (PMC10587178; doi:10.1038/s41598-023-44728-w)
Supplement: Supplementary file 1 — Supplementary Information. [file 41598_2023_44728_MOESM1_ESM.docx]

**Supplementary Materials**

**Structural Characterization, Biofunctionality, and Environmental Factors Impacting Rheological Properties of Exopolysaccharide Produced by Probiotic Lactococcus lactis C15**

**Gafar Bamigbade^1^, Abdelmoneim H. Ali^2^, Athira Subhash^1^, Camila Tamiello-Rosa^1^, Farah R. Al Qudsi^3^, Gennaro Esposito^4^, Fathalla Hamed****^5^, Shao-Quan Liu^6^, Ren-You Gan^7^, Basim Abu-Jdayil^8*^, Mutamed Ayyash^1^***

^1^ Department of Food Science, College of Agriculture and Veterinary Medicine, United Arab Emirates University (UAEU), Al Ain, UAE

^2^ Department of Food Science, Faculty of Agriculture, Zagazig University, Zagazig 44511, Egypt

^3^ Department of Nutrition and Food Technology, Jordan University of Science and Technology, Irbid 21121, Jordan

^4^ Science Division - New York University Abu Dhabi, NYUAD Campus, Saadiyat Island, PO Box 129188, Abu Dhabi, UAE

^5^ Department of Physics, College of Science, United Arab Emirates University (UAEU), PO Box 1555, Al Ain, UAE

^6^ Department of Food Science and Technology, Faculty of Science, National University of Singapore, Science Drive 2, Singapore 117542

^7^ Singapore Institute of Food and Biotechnology Innovation (SIFBI), Agency for Science, Technology and Research (A*STAR), Singapore 138669, Singapore

^8^ Chemical and Petroleum Engineering Department, College of Engineering, United Arab Emirates University (UAEU), PO Box 15551, Al Ain, UAE

***Corresponding authors:**

Mutamed Ayyash: [mutamed.ayyash@uaeu.ac.ae](mailto:mutamed.ayyash@uaeu.ac.ae)

Basim Abu-Jdayil: [babujdayil@uaeu.ac.ae](mailto:babujdayil@uaeu.ac.ae)

**NMR analysis**

*NMR experimental details*. The one-dimension (1D) ^1^H NMR and ^13^C NMR spectra and the two-dimension (2D) ^1^H -^1^H total correlation spectroscopy (TOCSY) ^1^ and ^13^C‒^1^H heteronuclear single quantum correlation (HSQC) ^2^ and heteronuclear multiple bond correlation (HMBC) ^3^ spectra were recorded in D_2_O at 298 K with a 18 mg mL^−1^ at 600.19 MHz and 150.92 MHz, respectively for ^1^H and ^13^C. The TOCSY experiment was run with a DIPSI spin-lock train ^4^ applied for 50 ms at γB_1_/2π = 10 kHz. Quadrature detection in the indirect dimension was obtained by TPPI method ^5^. Data were collected over a 12 ppm window using 2048 points in t_2_ and 400 in t_1_ with 64 scans/ t_1_ increment. The HSQC experiment was run with sensitivity enhancement using echo/antiecho-TPPI quadrature in the indirect dimension, trim pulses in INEPT transfer and gradients in retro-INEPT ^6-8^. Echo-antiecho-TPPI quadrature detection was employed also in HMBC. Heteronuclear spectra were collected over 12 ppm and 165 (or 220) ppm windows in the ^1^H and ^13^C dimensions, respectively, using 2048 points in t_2_ and 128 in t_1_ with 256 scans/ t_1_increment. For both homonuclear and heteronuclear spectra, prior to 2D Fourier transform, squared sine-bell apodization shifted by π/2 in either dimensions and linear prediction to double the data size in t_1_ were applied, with zero filling to obtain 2Kx1K matrices of reals.

Table S1. Molar ratio of the monosaccharides of EPS-C15.

| **S/N** | **Monosaccharides** | **Molar ratio** |
| --- | --- | --- |
| 1 | Arabinose | 2.0 |
| 2 | Xylose | 2.7 |
| 3 | Mannose | 1.0 |
| 4 | Glucose | 21.3 |

All monosaccharide analysis were done in triplicates.

Table S2: Regressed parameters of power-law model of EPS-C15 solutions.

| EPS solutions | pH | M | Ƞ | R^2^ |
| --- | --- | --- | --- | --- |
| CaCl_2_ | 4.0 | 0.0017 | 0.88 | 0.98 |
| CaCl_2_ | 6.0 | 0.0016 | 0.93 | 0.99 |
| NaCl | 4.0 | 0.0025 | 0.85 | 0.99 |
| NaCl | 6.0 | 0.0015 | 0.92 | 0.99 |
| Water | 4.0 | 0.0046 | 0.76 | 0.99 |
| Water | 6.0 | 0.0049 | 0.75 | 0.99 |

All rheological measurements and subsequent calculations were carried out in duplicates.

Table S3: Activation energy of flow for different EPS-C15 solutions.

| EPS solutions | pH | Ea (KJ/mol) |
| --- | --- | --- |
| CaCl_2_ | 4.0 | 33.7 |
| CaCl_2_ | 6.0 | 27.4 |
| NaCl | 4.0 | 14.8 |
| NaCl | 6.0 | 15.7 |
| H_2_O | 4.0 | 10.2 |
| H_2_O | 6.0 | 10.1 |

All rheological measurements and subsequent calculations were carried out in duplicates.

Figure S1: Molecular weight analysis of EPS-C15 by using gel permeation chromatography and refractive index.

Figure S2: Power law model (shear stress) of EPS-C15 in CaCl_2_ at pH 6.0 (◇), CaCl_2_ at pH 4.0 (☐), NaCl at pH 6.0 (○), NaCl at pH 4.0 (△), H_2_O at pH 6.0 (+), and H_2_O at pH 4.0 (✕). All rheological measurements were carried out in duplicates.

Figure S3: Amplitude sweep test of storage (G′, A) and loss (G′′, B) of EPS-C15 in CaCl_2_ at pH 6.0 (◇), CaCl_2_ at pH 4.0 (☐), NaCl at pH 6.0 (○), NaCl at pH 4.0 (△), H_2_O at pH 6.0 (+), and H_2_O at pH 4.0 (✕).

Figure S4: Viscoelastic properties of EPS-C15 in CaCl_2_ at pH 6.0 (◇), CaCl_2_ at pH 4.0 (☐), NaCl at pH 6.0 (○), NaCl at pH 4.0 (△), H_2_O at pH 6.0 (+), and H_2_O at pH 4.0 (✕).

Figure S5: Time-dependent behavior of EPS-C15 in CaCl_2_ at pH 6.0 (◇), CaCl_2_ at pH 4.0 (☐), NaCl at pH 6.0 (○), NaCl at pH 4.0 (△), H_2_O at pH 6.0 (+), and H_2_O at pH 4.0 (✕).

**References**

(1) Braunschweiler, L.; Ernst, R. R. Coherence transfer by isotropic mixing: Application to proton correlation spectroscopy. *Journal of Magnetic Resonance (1969)* **1983,** *53* (3), 521-528.

(2) Bodenhausen, G.; Ruben, D. J. Natural abundance nitrogen-15 NMR by enhanced heteronuclear spectroscopy. *Chem. Phys. Lett.* **1980,** *69* (1), 185-189.

(3) Cicero, D. O.; Barbato, G.; Bazzo, R. Sensitivity Enhancement of a Two-Dimensional Experiment for the Measurement of Heteronuclear Long-Range Coupling Constants, by a New Scheme of Coherence Selection by Gradients. *J. Magn. Reson.* **2001,** *148* (1), 209-213.

(4) Shaka, A. J.; Lee, C. J.; Pines, A. Iterative schemes for bilinear operators; application to spin decoupling. *Journal of Magnetic Resonance (1969)* **1988,** *77* (2), 274-293.

(5) Marion, D.; Wüthrich, K. Application of phase sensitive two-dimensional correlated spectroscopy (COSY) for measurements of 1H-1H spin-spin coupling constants in proteins. *Biochem. Biophys. Res. Commun.* **1983,** *113* (3), 967-74.

(6) Palmer, A. G.; Cavanagh, J.; Wright, P. E.; Rance, M. Sensitivity improvement in proton-detected two-dimensional heteronuclear correlation NMR spectroscopy. *Journal of Magnetic Resonance (1969)* **1991,** *93* (1), 151-170.

(7) Kay, L. E.; Keifer, P.; Saarinen, T. Pure absorption gradient enhanced heteronuclear single quantum correlation spectroscopy with improved sensitivity. *J. Am. Chem. Soc.* **1992,** *114* (26), 10663-10665.

(8) Schleucher, J.; Schwendinger, M.; Sattler, M.; Schmidt, P.; Schedletzky, O.; Glaser, S. J.; Sørensen, O. W.; Griesinger, C. A general enhancement scheme in heteronuclear multidimensional NMR employing pulsed field gradients. *J. Biomol. NMR* **1994,** *4* (2), 301-6.
